# Effect of flavor on neuronal responses of the hypothalamus and ventral tegmental area

**DOI:** 10.1038/s41598-019-47771-8

**Published:** 2019-08-02

**Authors:** A. M. van Opstal, A. A. van den Berg-Huysmans, M. Hoeksma, C. Blonk, H. Pijl, S. A. R. B. Rombouts, J. van der Grond

**Affiliations:** 1 Leiden University Medical Center, Department of Radiology, Leiden, The Netherlands; 2 Unilever Research & Development, Vlaardingen, The Netherlands; 3 Leiden University Medical Center, Department of Internal Medicine, Section Endocrinology, Leiden, The Netherlands; 4 Leiden Institute for Brain and Cognition (LIBC), Leiden, The Netherlands; 5 Institute of Psychology, Leiden University, Leiden, The Netherlands

**Keywords:** Neurology, Nutrition

## Abstract

Although it is well known that food intake is affected by the palatability of food, the actual effect of flavoring on regulation of energy-homeostasis and reward perception by the brain, remains unclear. We investigated the effect of ethyl-butyrate (EB), a common non-caloric food flavoring, on the blood oxygen level dependent (BOLD) response in the hypothalamus (important in regulating energy homeostasis) and ventral tegmental area (VTA; important in reward processes). The 16 study participants (18–25 years, BMI 20–23 kg/m^2^) drank four study stimuli on separate visits using a crossover design during an fMRI setup in a randomized order. The stimuli were; plain water, water with EB, glucose solution (50gram/300 ml) and glucose solution with EB. BOLD responses to ingestion of the stimuli were determined in the hypothalamus and VTA as a measure of changes in neuronal activity after ingestion. In the hypothalamus and VTA, glucose had a significant effect on the BOLD response but EB flavoring did not. Glucose with and without EB led to similar decrease in hypothalamic BOLD response and glucose with EB resulted in a decrease in VTA BOLD response. Our results suggest that the changes in neuronal activity in the hypothalamus are mainly driven by energy ingestion and EB does not influence the hypothalamic response. Significant changes in VTA neuronal activity are elicited by energy combined with flavor.

## Introduction

In understanding the complexity of eating behavior, understanding the regulation of energy balance by is essential. Energy balance and intake is regulated by the brain by two major regulatory systems: the homeostatic system and the reward systems^1–3^. The hypothalamus is known to control energy homeostasis through glucose and energy sensing and appetite regulation^1,3^. The hypothalamus has been shown to respond directly to the ingestion of glucose and plays a pivotal role in central glucose sensing^4–9^. This central glucose sensing and metabolism have been established as an essential part of control of feeding and hunger^10^. In addition to the hypothalamus, hunger and appetite are also controlled in conjunction with the orbitofrontal cortex, insula and the reward system^1,11^. The reward, or mesolimbic, system is responsible for the hedonic response to food. An important brain area involved in this hedonic response is the ventral tegmental area (VTA). The VTA forms the basis of dopamine signaling in the mesolimbic system, which is a key substrate for reward prediction and response^12^. Furthermore, the VTA is anatomically and functionally connected with the hypothalamus and integrates homeostatic signals with reward responses and therefore plays a pivotal role in regulating palatable feeding^13–15^. Indeed, the VTA has been shown to have a (reward) response to both taste stimuli and the ingestion of energy^8,9,16^.

Pleasantness of food, affected by factors such as flavor and texture, has a direct influence on feeding behavior by making food palatable and attractive and thereby eliciting a reward response in the brain^11,13,17^. In this respect, pleasant flavoring might cause overeating and cause an energy misbalance^18^. Flavor of food might also be involved in energy balance as flavor is an important part of the palatability of food and plays a role in satisfying and the rewarding effects and consumption volume of food^17,19^. In this respect, pleasantness of food enhances the satiating effect of high-fat and high-carbohydrate meals^20^. Additionally, flavor of food without ingestion of energy has been shown elicit responses from the brain and the periphery. Ingestion of a fat aroma with low fat content has been shown to elicit and modulate responses from the gustatory system^21^. Also, sweetness that is rated as pleasant without caloric intake has been shown to elicit an insulin response when applied only to oral cavity^22,23^. Although the evidence for the effect of taste on the cephalic phase insulin response is limited, these results do suggest that the flavor of food, independent of energy content, might influence the central regulation of the peripheral energy metabolism in addition to activation of the reward pathway in the brain. Furthermore, when glucose is paired with a congruent flavoring the perception of flavor is enhanced, and results in a greater feeling of satiety^24–26^. Therefore flavoring may have an effect on the responses in both the VTA and hypothalamus to glucose ingestion and energy ingestion in general. Additionally, the added perceived sweetness and satiation by flavoring is interesting to investigate as this might be used as a strategy towards reduction of energy content as less nutritive sweetener could be used to reach the same level of overall sweetness^26^.

Taken together, earlier research indicates that flavor may affect both homeostatic and hedonic aspects involved in energy intake. We aimed to gain a better understanding of the homeostatic and hedonic brain responses to flavoring and energy content separately and the combination of both stimuli. Therefore we investigated the changes in neuronal activity, as measured by BOLD response, to the ingestion of glucose, Ethyl-Butyrate (EB) a common fruity food flavoring, and a combination of both glucose and EB in the hypothalamus and VTA in normal weight young men. We hypothesized that flavoring with EB, with and without glucose, could affect the neuronal activity in the hypothalamus and VTA.

## Results

### Subject characteristics

Sixteen participants were enrolled in the study. All participants successfully completed the all study visits. Table 1 shows the subject characteristics of the study participants. All showed normal fasting glucose (3.9–5.5 mmol/L) and insulin levels (<20 mIU/L) and these levels were not different between visits.

**Table 1:** Subject characteristics.

	n = 16
Age (years)	20.6 ± 1.3
Height (m)	1.83 ± 0.05
Weight (kg)	72.4 ± 5.5
BMI (kg/m^2^)	21.7 ± 1.1
Glucose Fasted*	4.7 (4.2–5.4)
Post glucose ingestion*	6.9 (5.7–8.1)
Insulin Fasted*	6.5 (3.5–15.0)
Post glucose ingestion*	25.0 (9.5–80.0)

Values in mean ± standard deviation. Glucose and insulin levels in median and range. Glucose levels in mmol/L, normal fasted range: 3.9–5.5 mmol/L, Insulin levels in mU/L, normal fasted range: <20 mU/L. *Average fasted blood levels over all four visits and the average post ingestion levels for two visits with glucose stimuli per subject measured 30 minutes after ingestion.

### Hunger and flavor scores

Subjective VAS scores for feelings of hunger and for rating of flavor of the study stimuli are shown in Table 2. Before consumption, no significant differences were found between visits. The plain water and water flavored with EB stimuli initiated a significant increase in VAS scores for hunger. The flavor VAS score for water with EB was significantly lower compared with the other three study stimuli. The VAS score for flavor for the combined stimulus of glucose and EB was not significantly different from water and glucose stimuli but was rated significantly more pleasant than EB alone.

**Table 2:** Visual analogue scale (VAS) scores for hunger and flavor.

	Water	EB	Glucose	Glucose + EB
VAS hunger pre-ingestion	5.4 ± 2.3	5.8 ± 2.1	5.4 ± 2.2	5.9 ± 2.1
VAS hunger post-ingestion	6.6 ± 1.8	6.8 ± 1.6	5.5 ± 2.4	5.7 ± 2.3
Delta VAS hunger	1.2 ± 1.2	0.9 ± 1.6	0.2 ± 2.2	−0.3 ± 1.6
VAS flavor	5.2 ± 1.4	3.9 ± 1.5	5.4 ± 1.6	5.3 ± 2.5

Values in mean ± standard deviation. VAS consisted of a 10 cm line scored from 0 to 10, anchors for the VAS score for hunger 0: ‘not hungry’ and 10: ‘extremely hungry’, anchors for the VAS score for flavor 0: ‘very unpleasant’ and 10: ‘very pleasant’

### Hypothalamic activity changes

**Figure 1:**
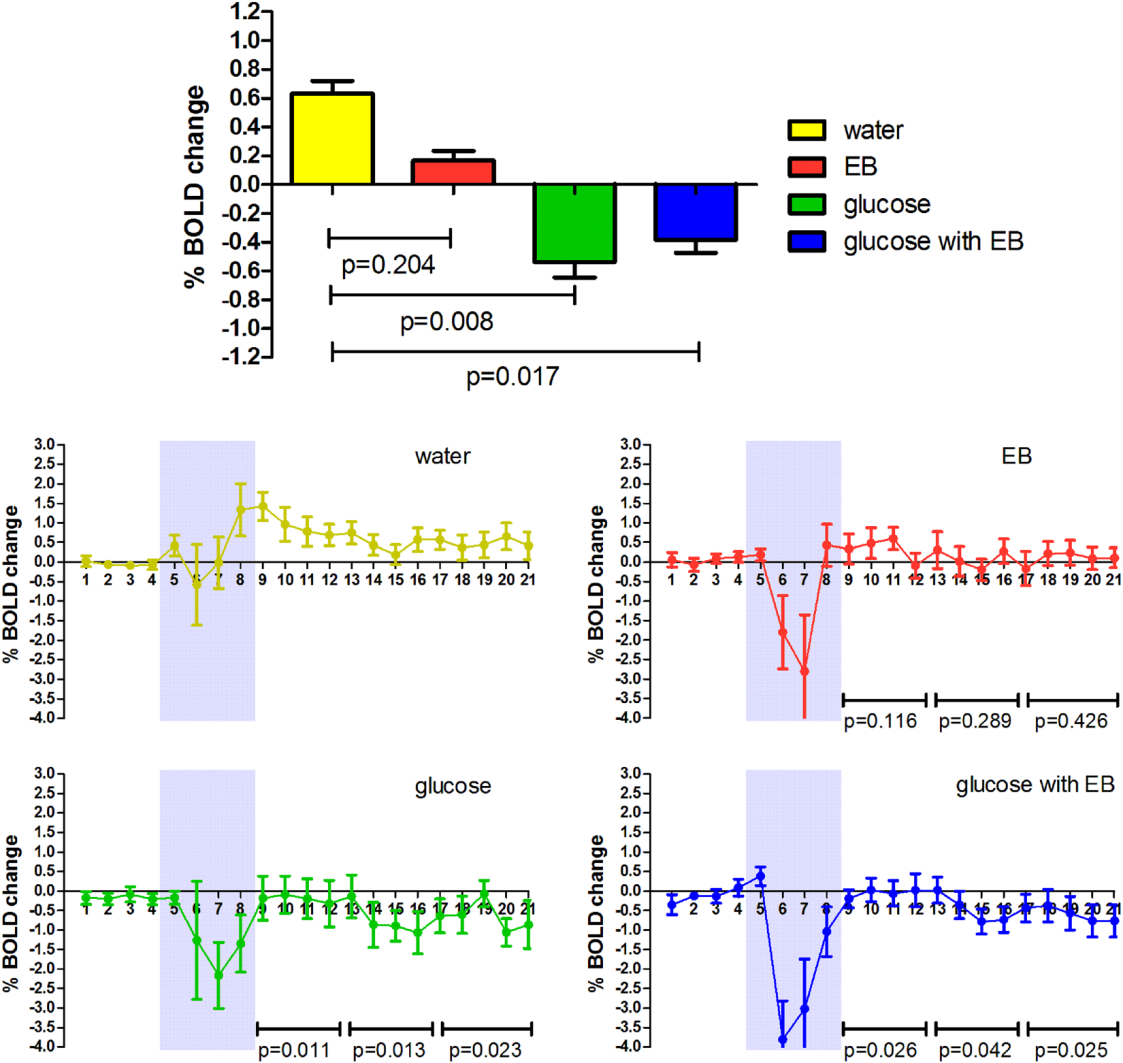
Hypothalamic mean BOLD signal change and BOLD time courses. BOLD responses to all four study stimuli (Mean % change with SEM). Percentage change from pre-ingestion (0–5 minutes) to post-ingestion (minute 9–21 post ingestion) were calculated. P-values in the top panel for the differences in percentage change between study stimuli were tested against the water intervention with linear mixed model analysis using the entire response. P-values in the bottom panel for differences in percentage change between study stimuli were tested against the water intervention with linear mixed model analysis per time segment (min 9–13, min 14–17 and min 18–21). Grey areas in the time course graphs indicate period during which the stimuli were consumed, data points were excluded from the analysis.

Mean hypothalamic BOLD responses per study stimulus are shown in Fig. 1 for illustration (top panel). Compared to baseline BOLD signal(before ingestion), ingestion of water led to a mean positive 0.63% BOLD response. Ingestion of water flavored with EB led to a mean positive BOLD response of 0.18%. Both water with glucose and water with glucose combined with EB led to a mean negative BOLD response of −0.54% and −0.38%, respectively.

When determining the effect of energy content and the presence of EB flavoring on the hypothalamic BOLD response we found that energy content had a significant effect on hypothalamic activity (p = 0.006), however the EB flavoring did not have a significant effect (p = 0.430). Energy content and EB flavoring did not have a significant (p = 0.310) interactive effect on the BOLD response, possibly due to the lack of effect of EB alone.

Compared to the plain water response, water flavored with EB did not lead to significant BOLD response (p = 0.204). On the contrary, both ingestion of glucose with and without EB resulted in a significant negative BOLD response (glucose only p = 0.008, and glucose flavored with EB p = 0.017). The addition of EB to glucose did not lead to significantly additive effect compared to glucose without flavoring (p = 0.870). Ingestion of glucose flavored with EB ingested at 0 °C did not lead to different response compared to ingestion at room temperature. When looking at the response over different time periods (Fig. 1 bottom panel, min 9–12, min 13–17 and min 18–21) we found that the response to glucose and glucose combined with EB was significantly different from water over all three time periods (p = 0.011, p = 0.013 and p = 0.023 for glucose and p = 0.026, p = 0.042 and p = 0.025 for glucose combined with EB).

### Ventral tegmental area activity changes

**Figure 2:**
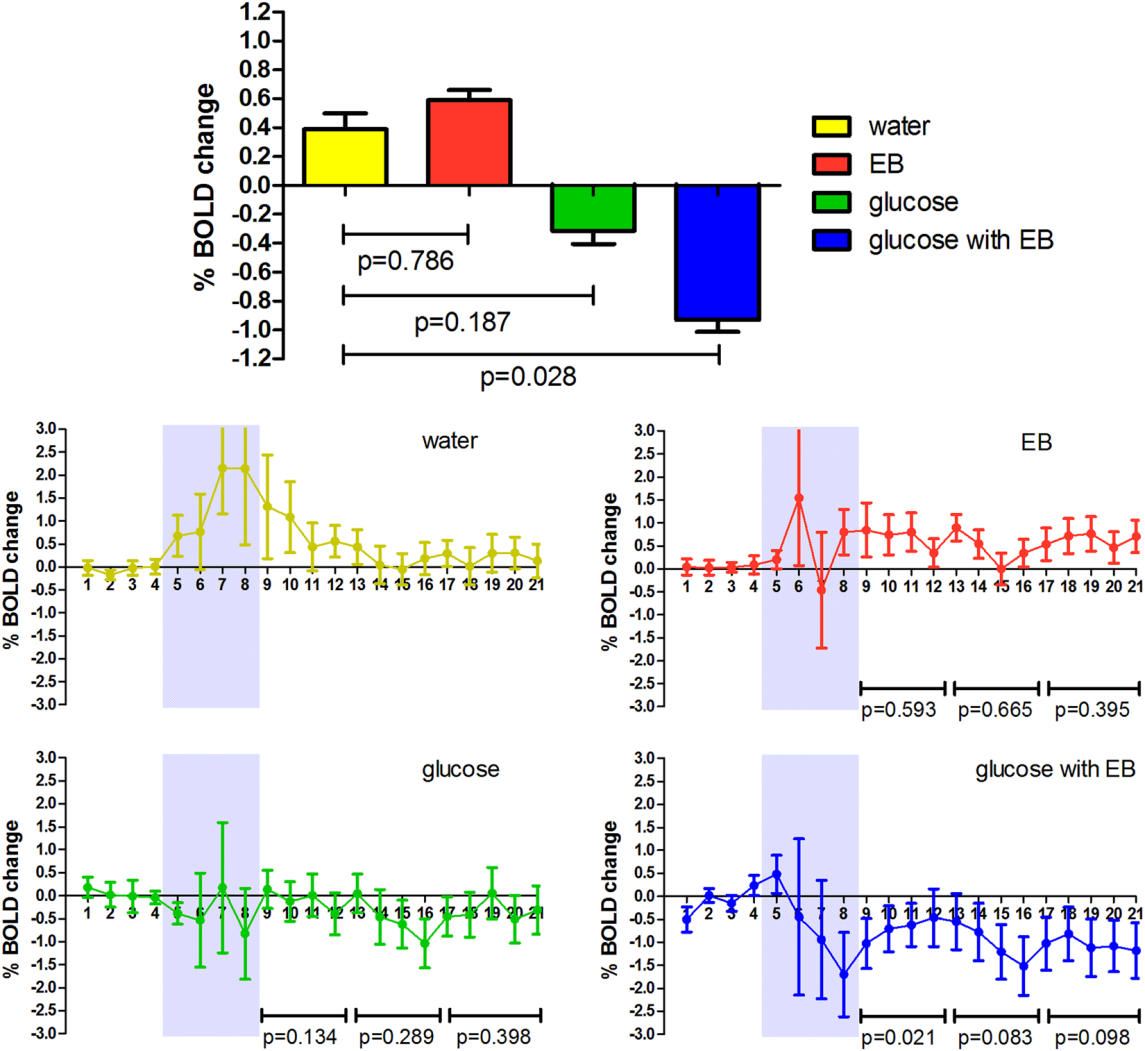
Ventral Tegmental Area (VTA) mean BOLD signal change and BOLD time courses. BOLD responses to all four study stimuli (Mean % change with SEM). Percentage change from pre-ingestion (0–5 minutes) to post-ingestion (minute 9–21 post ingestion) were calculated. P-values in the top panel for the differences in percentage change between study stimuli were tested against the water intervention with linear mixed model analysis using the entire response. P-values in the bottom panel for differences in percentage change between study stimuli were tested against the water intervention with linear mixed model analysis per time segment (min 9–13, min 14–17 and min 18–21). Grey areas in the time course graphs indicate period during which the stimuli were consumed, data points were excluded from the analysis.

Mean VTA BOLD responses per study stimulus are shown in Fig. 2 for illustration (top panel). Compared to baseline BOLD signal (before ingestion), ingestion of water led to a mean positive 0.38% BOLD response. Ingestion of water flavored with EB led to a mean positive BOLD response of 0.59%. Similar to the hypothalamic response, both water with glucose and water with glucose combined with EB led to a mean negative BOLD response of −0.31% and −0.93%.

When determining the effect of energy content and the presence of EB flavoring on the overall BOLD response we found that energy content had a significant effect on VTA activity (p = 0.007), however the EB flavoring did not have a significant effect (p = 0.612). Energy content and EB flavoring did not have a significant (p = 0.363) interactive effect on the BOLD response, possibly due to the lack of effect of EB alone.

Compared to the plain water response, water flavored with EB did not lead to an altered BOLD response (p = 0.786). Ingestion of glucose only, resulted in a non-significant decrease in BOLD response (p = 0.187). On the contrary, glucose flavored with EB mean difference led to a significant decrease in BOLD response (p = 0.028), although this BOLD response was not significantly stronger compared to glucose only (p = 0.317). Ingestion of glucose flavored with EB ingested at 0 °C did not lead to different response compared to ingestion at room temperature. When looking at the response over different time periods (Fig. 2 bottom panel, min 9–12, min 13–17 and min 18–21) we found that the response to glucose combined with EB was significantly different from water during the first minutes of the response (p = 0.021) but that this difference disappeared during the later minutes.

### Associations between blood values, subjective ratings of hunger and flavor and the hypothalamic and VTA activity changes

Mixed model analysis showed that increased ratings of hunger before stimulus ingestion were associated with a positive shift of the BOLD response in both the hypothalamus (+0.2%, p = 0.043) and VTA (+0.2%, p = 0.012). Moreover, ratings of hunger after stimulus ingestion were also associated with a positive shift of the BOLD response in both the hypothalamus (+0.2%, p = 0.042) and VTA (+0.03%, p = 0.001). Subjective rating of pleasantness of flavor did not have a significant association with the BOLD response in either the hypothalamus (p = 0.512) and VTA (p = 0.573). Blood glucose levels were not significantly associated with the BOLD response of the hypothalamus or VTA. Insulin levels after ingestion were positively associated with the BOLD response in the VTA (+0.2%, p = 0.009).

## Discussion

The results of this study show that in the VTA the combination of glucose with EB flavoring led to a significant decrease in neuronal activity, which was threefold stronger than to glucose alone. EB flavoring in plain water (no caloric content) did not have any effect on the response of the VTA. In the hypothalamus the ingestion of glucose, and not EB, was associated with changes in neuronal activity.

The perception of sweetness, with and without energy content, has been shown to elicit a response from the reward system^13,16^. Sweetness is regarded by the brain as a predictor of energy content of the ingested food or drink^16,27,28^. VTA is active in expectation of reward^13^, sweet flavoring could therefore lead to an increase in VTA activity because of the expectation of incoming energy content. Interesting in this context are non-nutritive sweeteners, which are increasingly used in foods and beverages, because they deliver sweet taste without any caloric content^29,30^. However, the flavoring we used in our study is not necessarily perceived as sweet on its own without the addition of glucose or another sweetener. Additionally, the EB stimulus was rated as less pleasant then the other stimuli and therefore might not have been rewarding by itself. This could explain why we did not find a significant response from the VTA, although the activity did appear to increase, with just the EB flavoring added to plain water^8,9,16^. Additionally, EB could have effects on other brain areas that we missed by measuring only the responses of the hypothalamus and VTA.

Adding flavoring to sugars has been shown to amplify the sweet taste and palatability which could increase the rewarding response^19,20^. Additionally, when glucose is paired with a congruent flavoring, the perception of flavor is also enhanced^24,25^, and could subsequently lead to a stronger reward response. This is in line with our finding that EB combined with glucose elicited the most pronounced response from the VTA. Furthermore, energy coupled with flavoring has been shown to lead to stronger subjective feelings of satiation and short term satiety compared to either stimulus separately^26^, which can also be linked to a stronger reward response by the brain. The strongest response to the combined stimulus in the VTA could be explained by the VTAs role in regulating palatable eating by integrating energy content with reward via the VTAs anatomical and functional connections with the hypothalamus^13–15^. The strongest response to the combined stimulus could also be explained by the fact that this stimulus could be the most intense perceptual stimulation that affects multiple sensory modalities including both gustatory and olfactory aspects but also trigeminal sensation. However, we did not measure perceptual intensity ratings of the stimuli so we cannot determine of this was the case. The strongest effect of combined flavoring and glucose on the VTA could indicate that added flavoring can be used as strategy towards reduction of energy content, as our results indicate that the response to energy content could be strengthened when combined with flavoring therefore allowing for a possible lower total energy content.

In addition to reward effects, flavor might affect the neuronal activity of the hypothalamus as sweet taste without caloric intake has been shown to elicit an insulin response^22^. However, other studies indicate that taste only elicits or modulates a hormonal response in combination with caloric content, as regulated by the hypothalamus^5,30,31^. Earlier studies by our group have shown that the ingestion of glucose solution leads to a decreased activity throughout the brain and specifically in the hypothalamus^8,9,32^. The results from the current study confirm once again that hypothalamic activity decreases after glucose ingestion and show that the driving force of the hypothalamic response is indeed the ingestion of energy and not the flavor indicated by the lack of response to flavoring without energy content. Our result are also concordant with the results of other previous studies showing that flavor without energy does not affect the hypothalamus^4–7^.

Our data show that stronger feelings of hunger, both before and after stimulus ingestion, had a positive effect on activity change adjusted for treatment in both the hypothalamus and VTA. This indicates that a stronger feeling of hunger lessened the decrease in activity in the hypothalamus. In a state of hunger, the brain - and specifically the hypothalamus and VTA - have been shown to have a high activity^5,6,13,33^, which could thus decrease after energy intake and with satiety. Our finding that a higher subjective score for hunger leads to a larger decrease in activity in the hypothalamus and the VTA support this theory. This suggests that these responses might reflect an objective evaluation of (perceived) energy content and reward.

A limitation of this study is the generalizability as we only investigated a relatively small sample of male volunteers and it can be expected that sex differences are present, since it is known that there are several sex-specific differences in energy metabolism^34^. Additionally, we only investigated lean subjects and earlier studies have shown different hypothalamic function in obesity^35^. A limitation of our study design was the use of water as a negative control. Water might not be a negative control as could elicit a response a response from the hypothalamus as fluid balance is also regulated by the hypothalamus. However, since all our stimuli contained or were dissolved in water we this effect would be found with all stimuli. We did not measure thirst in our participants and therefore we could not correct for the effect of this per study visit on a possible response of the hypothalamus to regulate fluid balance. Furthermore, our combined stimulus of EB with glucose is a very complicated stimulus with gustatory and olfactory effects. The combined stimulus could lead to various combined effects on that might be different from just the additive effects of glucose plus flavoring. Unfortunately our current design does not allow us the determine these effects. A strength of our study was our crossover study design, which allowed for a reliable within-subject comparison between interventions as participants were their own controls.

Taken together, our results suggest that the response of the VTA is strongest to a combination of both flavor and energy and that the hypothalamic response is mainly driven by energy ingestion. Although neither response seemed dependent on subjective ratings of pleasantness in the basal brain areas investigated here, the levels of hunger do influence the responses in these areas. This suggests that the responses by the hypothalamus and VTA might be mostly involved in subconscious rather the conscious regulation of satiety, appetite and feeding behavior and is mainly driven by energy demand and intake.

## Methods

### Study participants

Sixteen healthy, normal-weight men participated in our study. Participants were recruited via local advertisements and through the use of mailing lists. All participants were between the ages of 18 and 25 and had a body mass index (BMI) ranging from 20 to 23. Exclusion criteria were the following: a history of diabetes or disturbed glucose metabolism; any genetic, psychiatric, renal, hepatic or chronic disease; recent fluctuations in weight >3 kg; current smoking; current alcohol consumption >21 units/week and use of recreational drugs; recent blood donation or participation in other biomedical research (within the last 3 months); use of medication affecting glucose of lipid metabolism; contra-indications for MRI-scanning. The protocol was approved by the Medical Ethics Committee of the Leiden University Medical Center (LUMC) and registered at clinicaltrails.gov under number NCT03202342. All investigations have been conducted according to the principles expressed in the Declaration of Helsinki. Written informed consent was obtained from all participants after complete written and verbal description of the study was given.

### Experimental procedure

**Figure 3:**
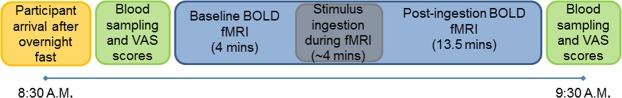
Study procedures. Illustrative depiction of the study procedures and timing during the study visits.

We used a randomized, controlled, crossover study design. A batch-wise randomization procedure, respecting 1^st^ order, was followed. The randomization was performed by an independent person. Participants arrived at MRI facilities of the Leiden University Medical Centre (LUMC) after an overnight fast on a separate study occasion for each stimulus. After blood sampling and recording pre scanning visual analogue scores (VAS), participants were positioned in the MRI scanner where the study stimuli were ingested within the scanner bore through a per oral tube during fMRI scanning. Study procedures are illustrated in Fig. 3. The different stimuli used and investigated were; plain water, water with ethyl-butyrate (EB), glucose solution and glucose solution with ethyl-butyrate (EB) (glucose solutions consisted of 50 grams of glucose dissolved in 300 ml water),stimuli were ingested at room temperature. Water was plain, non-chlorinated tap water. Ethyl-butyrate is a commonly used food flavoring with a fruity flavor and is an FDA and EU approved food flavoring under title 21 section 182.60 and EU Regulation 1334/2008 & 178/2002. EB was delivered in a liquid form dissolved in glycerol by Unilever Research and Development Vlaardingen B.V. EB and was first diluted to a lower concentration and then added to the 300 ml plain water or glucose solution to a concentration of 0.01%. The stimuli were tasted before the start of the study to determine the dose of EB that would be appropriate. Glucose samples were perceived to be sweetest. EB alone dissolved in water was perceived as slightly sweeter than water, but when combined with glucose did not enhance the sweetness when compared to glucose alone. EB alone as well as glucose alone were perceived to be fruity, and the combination of glucose with EB resulted in an enhancement of fruitiness. In the present trial, an additional fifth stimulus was investigated, consisting of a glucose solution with EB ingested at 0 °C, to investigate the effects of temperature. As the aim of the current study was to determine the effects of flavoring only and the effects of temperature have been described in an earlier study by our group^8^ the results of this condition are therefore only briefly shown and not discussed further in the current study. The stimuli were ingested five minutes after the start of the fMRI scan.

### Blood sampling and VAS scores

Blood samples were used to ascertain a normal glucose metabolism in each participant. On every test day, 2 blood samples (5 ml each) were drawn by venipuncture in an antecubital vein; one sample was taken before scanning and the other one after the scanning procedure, 30 minutes after ingestion of the study solution. Sample handling and analysis was performed by the laboratory for Clinical Chemistry at LUMC. Plasma glucose was measured using a fully automated Hitachi 704/911 system. Plasma insulin was measured by a Radio Immuno Assay (Medgenix, Fleurus, Belgium). A total amount of 10 ml blood was taken on each study day. Participants were asked to rate their feelings of hunger in advance of the scanning procedure and afterwards, using a visual analogue scale (VAS) which consisted of a 10 cm line, with ‘not hungry’ and ‘extremely hungry’ as anchors. Additionally, after the MRI scan pleasantness of the drink was rated using a similar VAS using ‘very unpleasant’ to ‘very pleasant’ as anchors.

### MRI data acquisition

The MRI was performed using a 3 Tesla whole body MRI scanner (Philips Achieva, Philips Healthcare, Best, The Netherlands) equipped with a 32-channel head coil. The protocol for structural MRI comprised a scout view for planning, a high resolution 3DT_1_-weighted sequence for registration purposes and a mid-sagittal high resolution single slice for accurate hypothalamus and VTA localization (repetition time 550 ms, echo time 10 ms, field of view 208 × 208 mm, voxel size = 0.52 × 0.52 × 14 mm). Mid-sagittal fMRI was performed for 21 minutes in total, by a T_2_* weighted, gradient echo-planar imaging (EPI) sequence mid-sagittal single slice that renders BOLD contrast (repetition time 120 ms; echo time 30 ms; flip angle 30°; field of view 208 × 208 mm; voxel size = 0.81 × 0.90 × 14 mm; 21 k-line excitations for each dynamic volume, scanning time of one dynamic image 2.52 seconds for a total of 500 data points). A slice thickness of 14 mm was chosen to encompass the hypothalamus in the left to right direction and a single slice technique was used for sufficient signal to noise ratio.

### High resolution mid brain functional MRI analysis

**Figure 4:**
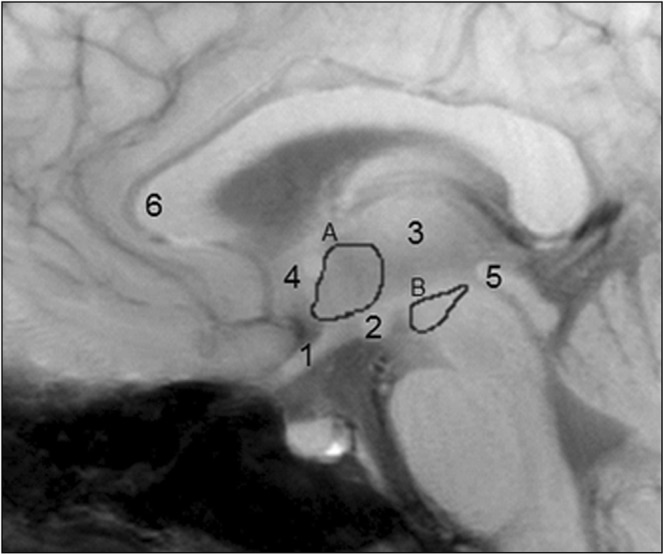
A representative sagittal view with the hypothalamus (A) and the VTA (B) ROIs. For (A), the optic chiasm (1), the mammillary bodies (2), the thalamus (3) and the anterior commissure (4) were used as landmarks. For (B), half the volume of (A), the top of the cerebral aqueduct (5) and the mammillary bodies (2) were used as landmarks. The reference ROI was drawn superior of the genu to the corpus callosum (6) in the grey matter.

Data was pre-processed as described previously^7^. In short data was averaged for each set of 4 subsequent volumes, reducing the 500 dynamic scans to 125 time points used for further analysis. Regions of interests (ROIs) were drawn manually, using subject-specific T_1_ images to define anatomical borders. Three ROIs were drawn: the hypothalamus, the VTA and a reference area. The ROI for the hypothalamus was drawn as described earlier using the optic chiasm, mammillary bodies, thalamus and anterior commissure as anatomical landmarks^7^. Using literature describing the VTA region^36,37^ we defined the ROI for the VTA in the midbrain using the top of the cerebral aqueduct and the mammillary bodies as anatomical landmarks (example ROIs shown in Fig. 4). To correct for scanner drift, a third internal reference ROI was selected superior to the genu of the corpus callosum in the grey matter. BOLD signals derived from the hypothalamus and the VTA were corrected for BOLD signals obtained from this reference ROI by dividing the ROI BOLD signals by the reference BOLD signal. Percentage BOLD change from corrected baseline was calculated by dividing the post ingestion BOLD signal by the average baseline BOLD signal for the individual post ingestion dynamic scans/measurement time points (n = 125) and for the total post ingestion period (minute 8–21 of the fMRI scan).

### Statistical analysis

Statistical analysis was performed using SPSS version 23. Differences in blood levels and VAS scores between pre- and post-ingestion and between study stimuli were tested with repeated measure ANOVA. All fMRI results are reported as BOLD change relative to baseline (0–5 minutes pre-drinking BOLD signal). To determine the main effect of energy content (presence of glucose yes or no) and flavor (presence of EB yes or no) on the study stimuli linear mixed model analysis was performed using energy content and the presence of flavoring and the study occasion as fixed effects and subject*study occasion as a random factor and the BOLD measurement time points as a covariate. To determine the interaction between energy content and flavoring the same mixed model was performed with an interaction term of both factors as a fixed effect. Statistical analysis of the difference in treatment effect between the study stimuli was performed by similar linear mixed model analysis using the study stimulus and study occasion as fixed effects, time point as a covariate and subject*occasion as a random factor using plain water as a negative and glucose solution as a positive reference stimulus. To determine the association between blood levels and the subjective ratings of hunger and flavor of the study stimulus and the BOLD response of the hypothalamus and VTA additional similar mixed models were used where these variables were added as covariates. All these mixed model analyses were applied on the n = 125 point dataset and therefore consider the entire response. To determine whether the difference in treatment effect changed during the measured time period we performed additional mixed model analysis over three time bins (min 9–12, min 13–17 and min 18–21).

## Data Availability

The raw data supporting the conclusions of this manuscript will be made available by the authors, without undue reservation, to any qualified researcher.
